# Structure-PPi: a module for the annotation of cancer-related single-nucleotide variants at protein–protein interfaces

**DOI:** 10.1093/bioinformatics/btv142

**Published:** 2015-03-11

**Authors:** Miguel Vázquez, Alfonso Valencia, Tirso Pons

**Affiliations:** Structural Biology and BioComputing Programme, Spanish National Cancer Research Centre (CNIO), 28029 Madrid, Spain

## Abstract

**Motivation:** The interpretation of cancer-related single-nucleotide variants (SNVs) considering the protein features they affect, such as known functional sites, protein–protein interfaces, or relation with already annotated mutations, might complement the annotation of genetic variants in the analysis of NGS data. Current tools that annotate mutations fall short on several aspects, including the ability to use protein structure information or the interpretation of mutations in protein complexes.

**Results:** We present the Structure–PPi system for the comprehensive analysis of coding SNVs based on 3D protein structures of protein complexes. The 3D repository used, Interactome3D, includes experimental and modeled structures for proteins and protein–protein complexes. Structure–PPi annotates SNVs with features extracted from UniProt, InterPro, APPRIS, dbNSFP and COSMIC databases. We illustrate the usefulness of Structure–PPi with the interpretation of 1 027 122 non-synonymous SNVs from COSMIC and the 1000G Project that provides a collection of ∼172 700 SNVs mapped onto the protein 3D structure of 8726 human proteins (43.2% of the 20 214 SwissProt-curated proteins in UniProtKB release 2014_06) and protein–protein interfaces with potential functional implications.

**Availability and implementation:** Structure–PPi, along with a user manual and examples, isavailable at http://structureppi.bioinfo.cnio.es/Structure, the code for local installations at https://github.com/Rbbt-Workflows

**Contact:** tpons@cnio.es

**Supplementary Information:** Supplementary data are available at *Bioinformatics* online.

## 1 Introduction

Predicting how single-nucleotide variant (SNV) alters the function of protein products is a topic of growing interest in genomics and bioinformatics (reviewed in Hecht *et al*., 2013). One of the key limitations of the current computational tools for the prediction of the impact of SNVs is that protein–protein interactions are poorly considered or completely ignored. This is surprising since we know that proteins work as part of protein complexes and interaction networks, and a number of databases with high-quality 3D structurally resolved protein interactome networks are available (Meyer *et al*., 2013; Mosca *et al*., 2013), and increasingly used to understand human genetic diseases (Guo *et al*., 2013; Nishi *et al*., 2013; Vidal *et al*., 2011; Wang *et al*., 2012; Yates and Sternberg, 2013; for a recent review about this topic, see Das *et al*., 2014). In spite of this, methods that allow a systematic analysis of SNVs, considering known functional residues in spatial contact with the mutation, and including full atom-level description of protein–protein interfaces, are not available.

Indeed, only a few methods, e.g. PMut (Ferrer-Costa *et al*., 2005), SNPeffect (Reumers *et al*., 2005), SNPs3D (Yue *et al*., 2006), PolyPhen-2 (Adzhubei *et al*., 2010), PoPMuSiC (Dehouck *et al*., 2011) and MuPIT (Niknafs *et al*., 2013) use full atom-level description of protein structures explicitly, but they do not include a detailed information about protein–protein interfaces as part of their algorithms (see Supplementary Table S1).

Here, we describe Structure–PPi that precisely analyzes mutations data in their 3D protein complex context. This module represents a significant improvement on existing tools in terms of: (i) ability to map mutations onto 3D structures of protein–protein complexes (experimental and homology-based), (ii) description of functional residues around the SNVs in protein–protein interfaces, additionally Structure–PPi implements a full annotation schema annotating post-translational modification sites, catalytic sites, binding sites residues, Pfam domains and prediction of damaging effects from state-of-the-art methods. Besides, the system selects a single reference sequence for each protein-coding gene (i.e. principal isoform), and provides information about cancer somatic mutations, and their corresponding tumor origin and histology. Since this work was submitted two papers dealing with the analysis of disease mutations at protein-protein interfaces have appeared (Mosca *et al*., 2015; Porta-Pardo *et al*., 2015).

## 2 Implementation and capabilities

### 2.1 Overview

**Fig. 1.**
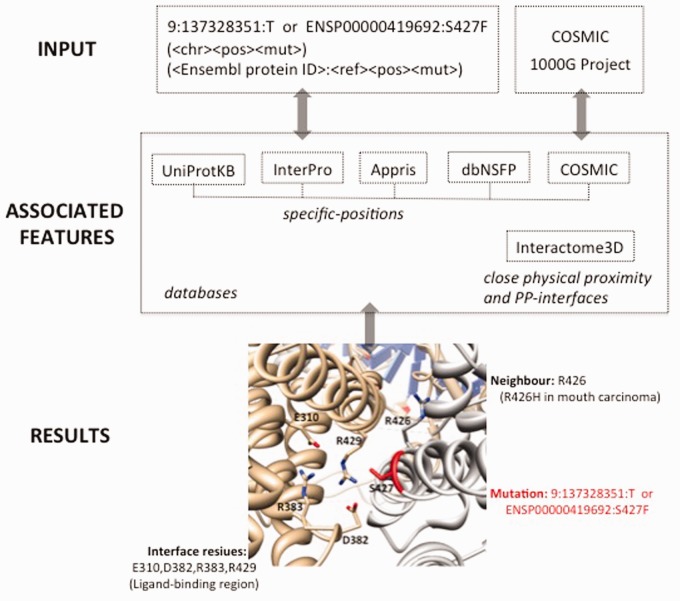
Flowchart of steps implemented in the Structure-PPi system (see Supplementary Table S2 for more details). The 3D protein complex interface between ENSP00000419692 and human Liver X nuclear receptor beta is also shown (PDB ID: 4nqa)

Structure–PPi offers a system to analyze SNV data in their protein 3D structure context. A genomic variant that leads to a substitution in a particular residue of a protein isoform is linked to features associated to that amino acid. Those features include secondary structure, post-translational modification sites, catalytic sites, binding sites residues, Pfam domains, signal peptides, trans-membrane regions, prediction of damaging effects with state-of-the-art methods (i.e. SIFT, Polyphen2, LRT, MutationTaster, MutationAssessor, FATHMM, VEST3, CADD) and somatic mutations extracted from: UniProt (UniProt Consortium, 2013), InterPro (Hunter *et al*., 2012), APPRIS (Rodriguez *et al*., 2013), dbNSFP (Liu *et al*., 2013) and COSMIC (Forbes *et al*., 2011). Residues in close physical proximity to query SNVs, are extracted from the corresponding 3D structures, including the experimental structures and homology-based models available in the Interactome3D (Mosca *et al*., 2013) database. The proximity information is used to generate annotations not directly affected by the investigated mutations but that could be disrupted by changes in the close vicinity (defaults 5 Å). Users may submit batches of tens of thousands SNVs to retrieve the available functional annotations for the corresponding SNVs and residues in spatial contact in that protein or the corresponding protein complex (Fig. 1). Figure 1 also shows the study of hotspot position S427 for the ENSP00000419692 protein isoform in bladder cancer. An assessment of Structure–PPi using a validation set (14 pathogenic and 10 neutral) in BRCA1 BRCT domains (Lee *et al*., 2010) is shown in Supplementary Table S3. Structure–PPi achieves a level of performance similar to that obtained by MetaSVM, a support vector machine algorithm, which incorporate results from state-of-the-art methods (i.e. SIFT, Polyphen2, MutationTaster, Mutation Assessor, FATHMM and LRT) and the maximum frequency observed in the 1000G project (Liu *et al*., 2013). The results are as follow: MetaSVM (accuracy: 0.83, recall: 1.00, precision: 0.78, MCC: 0.68) and Structure–PPi (accuracy: 0.88, recall: 0.79, precision: 1.00, MCC: 0.78). This assessment reveals that Structure–PPi shows a better precision than MetaSVM, and also a good agreement between predictions and observations. In addition, Supplementary Table S3 shows the utility of Structure–PPi for providing complementary information to the prediction methods. Indeed, this complementary information facilitates discrimination of false-positive results, and also identifies mutations that should be study in more details.

### 2.2 Coverage

Structure–PPi maps SNVs onto the protein 3D structure for 8726 human proteins (43.2% of the 20214 SwissProt-curated proteins in UniProtKB release 2014_06). This value of 43.2% is well above the 18% coverage reported by MuPIT (Niknafs *et al*., 2013).

### 2.3 Software implementation and requirements

Structure–PPi is an independent component of the Rbbt-framework (“Ruby bioinformatics toolkit” Rbbt; https://github.com/mikisvaz/rbbt; Vázquez *et al*., 2010). Structure–PPi runs on Unix-based systems (including Linux and Mac). Structure–PPi can be accessed by programmatic access for ruby developers, command-line mode for power users or HTML interface through a web browser (http://structureppi.bioinfo.cnio.es/Structure) for standard users. Structure–PPi includes a pair-wise alignment (Smith–Waterman) step to resolve any potential inconsistency between the isoform sequence and the sequence in the 3D structure, or differences between the isoform sequence and the reference UniProt. Depending on the database used, the throughput on a single process is around hundreds or thousands per second, with less than 500 MB of memory use. We will continue to develop the Structure–PPi, in particular its method for parallelizing file archival and retrieval, and software portability, to further facilitating inclusion into extended genome annotation workflows. We have pre-computed annotations for all coding nsSNVs in COSMIC v69 (∼741 276), and in 1000G Project (∼285 846). The results are available through the website, and some summary statistics and discussion of variants at protein interfaces can be found in Supplementary Table S4. This preliminary analysis might identify disruption of important interactions and improve our understanding about human diseases. Structure–PPi is currently used in different projects, including the ICGC-CLL analysis.

## 3 Conclusion

We present Structure–PPi, a system to facilitate the comprehensive analysis of cancer-related SNVs, which combines 3D protein structures of protein complexes with functional annotations from different databases. The system implements the generally accepted idea that strong indicators of positive selection for tumorigenesis (driver mutations) are located in functional domain/sites or they affect amino acid residues that have been shown to be important by 3D protein structure. Furthermore, the system provides information about known functional-residues in close physical proximity to query SNVs. Thus, Structure–PPi can provide both mechanistic and biological insights into the role of SNVs in a given cancer.

## Supplementary Material

Supplementary Data
