# Supplementary material for: Structure-PPi: a module for the annotation of cancer-related single-nucleotide variants at protein–protein interfaces
Source: Bioinformatics. 2015 Mar 11;31(14):2397–9. doi: 10.1093/bioinformatics/btv142 (PMC4495296; doi:10.1093/bioinformatics/btv142)
Supplement: Supplementary Data [file supp_31_14_2397__index.html]

Structure-PPi: a module for the annotation of cancer-related single-nucleotide variants at protein–protein interfaces — Structure-PPi: a module for the annotation of cancer-related single-nucleotide variants at protein–protein interfaces — Supplementary Data 

# Structure-PPi: a module for the annotation of cancer-related single-nucleotide variants at protein–protein interfaces

## Supplementary Data

files

**Files in this Data Supplement:**

- Supplementary Data - pdf file
