# Supplementary material for: Structure-PPi: a module for the annotation of cancer-related single-nucleotide variants at protein–protein interfaces
Source: Bioinformatics. 2015 Mar 11;31(14):2397–9. doi: 10.1093/bioinformatics/btv142 (PMC4495296; doi:10.1093/bioinformatics/btv142)
Supplement: Supplementary Data [file supp_btv142_manuscript-Vazquez_et_al_2014_suppl_R3.pdf]

Supplementary Information

Structure-PPi: a module for the annotation of cancer-related single-nucleotide variants at protein-protein interfaces.

Miguel Vazquez, Alfonso Valencia, and Tirso Pons
Structural Biology and BioComputing Programme. Spanish National Cancer Research Centre (CNIO), 28029 Madrid.

Table S1. Comparison of the Structure-PPi module with similar available tools.

	**Rbbt-Structure**	**MuPIT interactive**	**LS-SNP/PDB**	**MutDB**	**SNPs3D**	**LS-SNP**	**MutationAssessor**	**Polyphen-2**
Website URL	structureppi.bioinfo.cnio.es	mupit.icm.jhu.edu	ls-snp.icm.jhu.edu/ls-snp-pdb	mutdb.org	snps3d.org	modbase.compbio.ucsf.edu/LS-SNP	mutationassessor.org	genetics.bwh.harvard.edu/pph2
Status (Last Update)	(21-Nov-2014)	(xx-yyy-2013)	(xx-yyy-2012)	(06-Feb-2007)	(29-Oct-2008)	(21-Jun-2006)	(12-Sep-2012)	(08-Mar-2012)
Custom input? (single or batch query)	Yes. Accepts user genomic or protein coordinates	Yes. Accepts user genomic coordinates	Yes. Accepts user gene name, genomic coordinates, and identifiers from dbSNP, UniProtKB, KEGG and PDB	Yes. Accepts user gene or protein names, and identifiers from dbSNP and UniProtKB/Swiss-Prot variants (humsavar)	Yes. Accepts user dbSNP or RefSeq identifiers	Yes. Accepts user gene name, genomic coordinates, and identifiers from dbSNP, UniProtKB, and KEGG	Yes. Accepts user genomic or protein coordinates	Yes. Accepts user genomic or protein coordinates
Batch entry of variants	Yes (high-throughput)	Yes (high-throughput)	Yes	No	Yes	Yes	Yes (high-throughput)	Yes (high-throughput)
Interface	Browser-based, REST web service, and command-line	Browser-based	Browser-based	Browser-based	Browser-based	Browser-based	Browser-based	Browser-based
3D repository	PDB and Interactome3D	PDB	PDB	PDB	PDB	PDB+ 3D models[(1)]	PDB	PDB
Proximity to known functional residues considered?	Yes	Yes	Yes	No	No	Yes	Yes	Yes
User-parametrizable distance?	Yes	No	No	No	No	No	No	Yes
Annotation sources	UniProtKB/Swiss-Prot[e], APPRIS, Firestar, InterPro[f], COSMIC, dbNSFP, Interactome3D	UniProtKB/Swiss-Prot[e]	DSSP[a], DELPHI[b] KEGG, PDB	UniProtKB/Swiss-Prot[e], dbSNP, PDB,	PDB, dbSNP, OMIM, HGMD, KEGG, GO, UniProtKB/Swiss-Prot, PubMed	PDB, dbSNP, KEGG, PIBASE	UniProtKB/Swiss-Prot[e], COSMIC, Pfam[f], PDB, Piana, dbSNP	UniProtKB/Swiss-Prot[e], PDB, DSSP[a]
Download results option	Yes	No	No	No	Limited to Rasmol	Limited to Rasmol	Yes	Limited to WHESS
Quick access to pre-computed datasets	Yes	No	No	Yes	Yes	Yes	No	Yes (WHESS)

[a]Solvent accessibility, [b]Electrostatic surface potential, [d], [e]Feature table section, [f]Protein domains, [g]WHESS: whole human exome sequence space dataset, [(1)]3D models of single proteins but not protein-complexes, Piana: Protein-protein interaction database.

Table S2. List of functionalities implemented in the Structure-PPi module.

Tasks	Description
ANNOTATE	Annotates genomic mutations based on the protein features that are overlapping amino-acid changes.
ANNOTATE_MI	Annotates mutated isoforms based on the protein features that are overlapping amino-acid changes.
ANNOTATE_NEIGHBOURS	Annotates genomic mutations based on the protein features that are in close physical proximity to amino-acid changes.
ANNOTATE_MI_NEIGHBOURS	Annotates mutated isoforms based on the protein features that are in close physical proximity to amino-acid changes.
INTERFACES	Find variants that affect residues in protein-protein interaction interfaces. It uses the PDB files of protein-protein complexes annotated in the Interactome3d database (release 2014_1).
MI_INTERFACES	Find mutated isoforms with affected residues in protein-protein interaction interfaces.
MI_NEIGHBOURS	Find residues within physical proximity to amino-acid changes in mutated isoforms.
NEIGHBOUR_MAP	For a given PDB file, find all pairs of residues that fall within a given 'distance of each other. It uses the PDB files of individual proteins annotated in the Interactome3d database (release 2014_1).
NEIGHBOURS_IN_PDB	Use a PDB file to find the residues neighbouring, in three-dimensional space, a particular residue in a given sequence.
PDB_ALIGNMENT_MAP	Find the correspondence between sequence positions in a PDB file and in a given sequence. The PDB positions are reported as 'chain:position'.
PDB_CHAIN_POSITION_IN_SEQUENCE	Translate the positions of amino acids in a particular chain of the provided PDB file into positions inside a given sequence.
SEQUENCE_POSITION_IN_PDB	Translate the positions inside a given amino-acid sequence to positions in the sequence of a PDB file by aligning them.
Wizard	Retrieve all annotations, including neighbors and interfaces, by using genomic mutation, mutated isoform, or an identifier such as associated gene name or gene symbol.

We illustrate the performance and usefulness of the Structure-PPi module by applying this tool to a validation set of mutations (14 pathogenic and 10 neutral) defined in Lee et al., 2010. Mutations included in the validation set (Table 1 and Supplementary Table S1 in Lee et al., 2010) were classified by genetic or integrative methods that used a combination of data from different sources: co-occurrence with known deleterious mutations, personal and family history of patients carrying the variant, and co-segregation of the variant with disease within pedigrees. As you can see below, Structure-PPi achieves a level of performance similar to that obtained by MetaSVM, a support vector machine algorithm, which incorporate results from state-of-the-art methods (e.g., SIFT, PolyPhen-2, MutationTaster, Mutation Assessor, FATHMM, and LRT) and the maximum frequency observed in the 1000G project (for details see dbNSFP v2.8 database at https://sites.google.com/site/jpopgen/dbNSFP). In addition, Table S3 shows the utility of Structure-PPi for providing complementary information to the prediction methods. Indeed, this complementary information facilitates discrimination of false positive results (bold letters in the column MetaSVM), and also identifies mutations that should be study in more details (bold letters in the column StructurePPi).

For the purpose of comparison, we assume that the Structure-PPi annotations support a "(D)eleterious" prediction in the following two scenarios: *i*) "mutations in protein-protein interfaces" AND "mutation position" OR "its neighboring residues" accommodate variants in human diseases, and *ii*) "mutations outside protein-protein interfaces" AND "mutation position" AND "its neighboring residues" accommodate variants in human diseases. Otherwise, Structure-PPi suggests a careful experimental study of the mutations.

Despite the goal of Structure-PPi is to annotate mutations instead to predict damage, based on the previous assumptions we calculated the Accuracy, Recall (or Sensitivity), Precision, and Matthews Correlation Coefficient (MCC). Hereafter, we will refer to the following abbreviations: True positives (TP), correctly predicted disease-associated mutations. False positives (FP), neutral mutations predicted as disease ones. True negatives (TN), correctly predicted neutral mutations. False negatives (FN), disease-associated mutations predicted as neutral. Accuracy accounts for the fraction of mutations correctly predicted in function of the total number of mutations. Recall, also referred to as sensitivity by other authors, accounts for the proportion of correctly predicted disease-associated mutations in function of all the disease-associated mutations in the dataset. Precision accounts for the proportion of correctly predicted disease-associated mutations with respect to all the predicted disease-associated mutations. The Accuracy, Recall, Precision, and MCC were calculated according to the following formulas:

$$\text{Accuracy} = \frac{TP + TN}{TP + FP + TN + FN} \quad ; \quad \text{Precision} = \frac{TP}{TP + FP}$$

$$\text{Recall} = \frac{TP}{TP + FN} \quad ; \quad MCC = \frac{TP \cdot TN - FP \cdot FN}{\sqrt{(TP + FP)(TP + FN)(TN + FP)(TN + FN)}}$$

The results are as follow: MetaSVM (Accuracy: 0.83, Recall: 1.00, Precision: 0.78, MCC: 0.68) and Structure-PPi (Accuracy: 0.88, Recall: 0.79, Precision: 1.00, MCC: 0.78). This assessment reveals that Structure-PPi shows a better precision than MetaSVM, and also a good agreement between predictions and observations.

Table S3. Structure-PPi report of validated variants in BRCA1 BRCT domains.

**Pathogenic**	**UniProt Features (position)**	**UniProt Features (neighbors)**	**PPi**	**MetaSVM**	**StructurePPi**
T1685I	DOMAIN(BRCT1 - 1642:1736); VAR_063902(T->A)[a]; VAR_063903 (T->I)[b]	M1652I (polymorphism)	no	0.76(D)	**(T)***
T1685A	DOMAIN(BRCT1 - 1642:1736); VAR_063902(T->A)[a]; VAR_063903 (T->I)[b]	M1652I (polymorphism)	no	0.73(D)	**(T)***
M1689R	DOMAIN(BRCT1 - 1642:1736); VAR_063904(M->R)[a]; STRAND(1686:1689)	S1655A, K1702M (abolishes interaction with BRIP1); C1697R (ovarian cancer); S1715R, K1690Q (breast cancer); V1713G (polymorphism)	yes	0.26(D)	(D)
R1699W	DOMAIN(BRCT1 - 1642:1736); VAR_020703(R->W)[c]	C1697R (ovarian cancer)	yes	0.93(D)	(D)
G1706E	DOMAIN(BRCT1 - 1642:1736); VAR_063905(G->E)[a]; HELIX(1701:1708)	A1708E (abolishes ACACA binding in breast cancer); V1713G (polymorphism); K1702M (abolishes interaction with BRIP1)	no	0.80(D)	(D)
A1708E	DOMAIN(BRCT1 - 1642:1736); VAR_007796(A->E)[d]; HELIX(1701:1708)	L1786P (breast & ovarian cancer; G1706E (breast cancer)	no	0.85(D)	(D)
S1715R	DOMAIN(BRCT1 - 1642:1736); VAR_063906(S->R)[a]; STRAND(1712:1715)	M1689R (breast cancer); D1692N (ovarian cancer); V1713G (polymorphism)	no	0.37(D)	(D)
G1738R	VAR_063907(G->R)[a]; MUTAGEN(G->E, abolishes interaction with BRIP1)	C1697R (ovarian cancer)	yes	0.71(D)	(D)
L1764P	DOMAIN(BRCT2 - 1756:1855); VAR_063908 (L->P)[a]	I1766S, G1788V (breast cancer)	yes	0.63(D)	(D)
I1766S	DOMAIN(BRCT2 - 1756:1855); VAR_063909 (I->S)[a]; STRAND(1765:1768)	L1764P (breast cancer)	no	0.73(D)	(D)
M1775R	DOMAIN(BRCT2 - 1756:1855); VAR_063212 (M->K)[e]; VAR_007799 (M->R)[f]; STRAND(1773:1775)	P1776S (ovarian cancer)	yes	0.69(D)	(D)
M1775K	DOMAIN(BRCT2 - 1756:1855); VAR_063212 (M->K)[e]; VAR_007799 (M->R)[f]; STRAND(1773:1775)	P1776S (ovarian cancer)	yes	0.69(D)	(D)
G1788V	DOMAIN(BRCT2 - 1756:1855); VAR_063212 (G->V)[a]	L1764P (breast cancer); L1786P (breast & ovarian cancer)	no	1.07(D)	(D)
V1838E	DOMAIN(BRCT2 - 1756:1855); HELIX(1835:1844)	-	yes	0.86(D)	**(T)**
**Neutral**					
M1652I	DOMAIN(BRCT1 - 1642:1736); VAR_007795(M->I)[g]; STRAND(1651:1656)	V1665M (polymorphism)	yes	-0.85(T)	(T)*
M1652T	DOMAIN(BRCT1 - 1642:1736); VAR_007795(M->I)[g]; STRAND(1651:1656)	V1665M (polymorphism)	yes	-0.05(T)	(T)*
F1662S	DOMAIN(BRCT1 - 1642:1736); HELIX(1659:1672); VAR_052080(F->C)[h]	V1665M (polymorphism)	yes	-0.81(T)	(T)*
A1669S	DOMAIN(BRCT1 - 1642:1736); HELIX(1659:1672)	M1652I, V1665M (polymorphism)	yes	**0.35(D)**	(T)*
E1682K	DOMAIN(BRCT1 - 1642:1736)	-	yes	**0.73(D)**	(T)
T1720A	DOMAIN(BRCT1 - 1642:1736); HELIX(1717:1724); T->A (No effect on in vitro phosphorylation)	-	no	-0.50(T)	(T)
V1736A	DOMAIN(BRCT1 - 1642:1736)	G1738R, M1689R, S1715R (breast cancer); C1697R, P1749R (ovarian cancer); G1738E (abolishes interaction with BRIP1); V1713G (polymorphism); P1749R (reduces BRIP1 binding)	no	**0.85(D)**	(T)
R1751Q	HELIX(1748:1753)	P1749R (ovarian cancer & reduces BRIP1 binding); S1755A (No effect on in vitro phosphorylation)	no	**0.60(D)**	(T)
V1804D	DOMAIN(BRCT2 - 1756:1855)	-	no	-0.83(T)	(T)
P1859R	-	-	no	-0.41(T)	(T)

PPi: indicates mutations localized in a Protein-Protein interface (yes) or outside the interface (no); MetaSVM: score and predictions in parenthesis, extracted from dbNSFP database (https://sites.google.com/site/jpopgen/dbNSFP); StructurePPi: supporting annotations for mutations classified as "(D)eleterious" or "(T)olerated" (see details in the text); [a]Unknown pathological significance in Breast cancer. [b]Could be associated with cancer susceptibility; multifactorial likelihood analysis provides evidence for pathogenicity. [c]Observed in ovarian cancer. [d]Abolishes ACACA binding in Breast cancer. [e]Strongly reduced transcription transactivation; abolishes interaction with BRIP1 and RBBP8 in Breast cancer. [f]Alters protein stability and abolishes ACACA and BRIP1 binding in Breast cancer. [g]Rare polymorphism (dbSNP:rs1799967). [h]Polymorphism (dbSNP:rs28897695). *Note that variants M1652I, M1652T, and F1662S, that have been included as neutral in the validation data set of Lee et al. (2010), are variants with an uncertain clinical significance according to updated annotations in databases (i.e. increased risk of ovarian cancer). In addition, these variants affect protein-protein interfaces. The new annotations in databases are in agreement with the Structure-PPi feature score available on the website in the section "Damage predictions". The Structure-PPi feature score considers mutations in protein-protein interaction surfaces, COSMIC samples with mutations over the same residue, UniProt variants and their potential association with disease, and UniProt features critical for protein function and having a prevalence in COSMIC that is more than double than that in 1000 Genomes Project: MUTAGEN, DISULFID, DNA_BIND, METAL, INTRAMEM, CROSSLNK.

Table S4. General description of coding nsSNVs in COSMIC and 1000 Genomes Project.

**COSMIC**	**not_PPi**		**PPi**		**COSMIC**
Features	***Freq***	%	***Freq***	%	%PPi/%not_PPi
BINDING	369	0.05	54	0.35	6.54
CA_BIND	406	0.06	41	0.27	4.51
METAL	360	0.05	59	0.39	7.32
NP_BIND	1766	0.26	245	1.60	6.20
DNA_BIND	3883	0.57	537	3.51	6.18
MUTAGEN	1195	0.17	239	1.56	8.93
SITE	183	0.03	30	0.20	7.32
ACT_SITE	199	0.03	16	0.10	3.59
Firestar_Cat	342	0.05	51	0.33	6.66
Firestar_Bind	10083	1.48	699	4.57	3.10
MOD_RES	1606	0.24	147	0.96	4.09
CARBOHYD	718	0.11	11	0.07	0.68
LIPID	44	0.01	3	0.02	3.04
CROSSLNK	45	0.01	18	0.12	17.86
DISULFID	1422	0.21	71	0.46	2.23
VARIANT	9137	1.34	1091	7.13	5.33
HELIX	22072	3.23	4794	31.33	9.70
STRAND	15561	2.28	2834	18.52	8.13
TURN	2298	0.34	545	3.56	10.59
DOMAIN	121962	17.85	5135	33.56	1.88
TOPO_DOM	124880	18.27	2125	13.89	0.76
MOTIF	1291	0.19	198	1.29	6.85
REPEAT	28507	4.17	689	4.50	1.08
ZN_FING	14079	2.06	153	1.00	0.49
COILED	15873	2.32	219	1.43	0.62
COMPBIAS	20669	3.02	86	0.56	0.19
INTRAMEM	274	0.04	12	0.08	1.96
TRANSMEM	28979	4.24	118	0.77	0.18
Appris_Membr	1298	0.19	0	-	-
INIT_MET	96	0.01	2	0.01	0.93
NON_TER	1	0.00	0	-	-
SIGNAL	3668	0.54	1	0.01	0.01
Appris_Signal	2477	0.36	3	0.02	0.05
PROPEP	3508	0.51	20	0.13	0.25
TRANSIT	601	0.09	4	0.03	0.30
PEPTIDE	474	0.07	46	0.30	4.33
REGION	46921	6.87	2343	15.31	2.23
Tot_mutations	**683396**		**15303**		

**1000G**	**not_PPi**		**PPi**		**1000G**
Features	***Freq***	%	***Freq***	%	%PPi/%not_PPi
BINDING	70	0.03	6	0.17	6.21
CA_BIND	101	0.04	21	0.61	15.06
METAL	52	0.02	4	0.12	5.57
NP_BIND	369	0.15	33	0.95	6.48
DNA_BIND	446	0.18	14	0.40	2.27
MUTAGEN	231	0.09	25	0.72	7.84
SITE	38	0.02	5	0.14	9.53
ACT_SITE	26	0.01	4	0.12	11.14
Firestar_Cat	69	0.03	2	0.06	2.10
Firestar_Bind	2146	0.85	88	2.54	2.97
MOD_RES	440	0.18	9	0.26	1.48
CARBOHYD	356	0.14	4	0.12	0.81
LIPID	11	0.00	0	-	-
CROSSLNK	13	0.01	1	0.03	5.57
DISULFID	240	0.10	11	0.32	3.32
VARIANT	23153	9.21	392	11.30	1.23
HELIX	6305	2.51	1115	32.13	12.81
STRAND	4517	1.80	626	18.04	10.04
TURN	651	0.26	96	2.77	10.68
DOMAIN	36195	14.40	965	27.81	1.93
TOPO_DOM	40217	16.00	473	13.63	0.85
MOTIF	359	0.14	15	0.43	3.03
REPEAT	9297	3.70	156	4.50	1.22
ZN_FING	3778	1.50	26	0.75	0.50
COILED	6437	2.56	64	1.84	0.72
COMPBIAS	8294	3.30	11	0.32	0.10
INTRAMEM	39	0.02	2	0.06	3.71
TRANSMEM	9465	3.77	46	1.33	0.35
Appris_Membr	391	0.16	1	0.03	0.19
INIT_MET	36	0.01	0	-	-
NON_TER	0	-	0	-	-
SIGNAL	1837	0.73	0	-	-
Appris_Signal	1272	0.51	2	0.06	0.11
PROPEP	1308	0.52	8	0.23	0.44
TRANSIT	472	0.19	0	-	-
PEPTIDE	209	0.08	18	0.52	6.24
REGION	16768	6.67	500	14.41	2.16
Tot_mutations	**251297**		**3470**		

Features: UniProt key names in the "Feature Table" line; Freq: number of nsSNV in a feature; %: percentage of nsSNV in a feature respect to the total number of nsSNV in the dataset; %PPi/%not_PPi: indicates how frequent is a feature at protein-protein interfaces or outside them; not_PPi: nsSNV outside protein-protein interfaces; PPi: nsSNV in protein-protein interfaces; Tot_mutations: total number of nsSNV in the dataset; Firestar_Cat: Catalytic site residues ("Cat_Site_Atl") predicted by Firestar; Firestar_Bind: Binding site residues predicted by Firestar; Appris_Membr: a "Damaged" transmembrane helix predicted by the THUMP method implemented in Appris; Appris_Signal: a "Signal peptide" region predicted by the CRASH method implemented in Appris.

This preliminary analysis suggests that a large proportion of coding nsSNV is positioned in functional domains and in secondary structural regions, both in COSMIC and in 1000 Genomes Project (1000G). In addition, we observe an enrichment of features like VARIANT (sequence variations), MOD_RES (posttranslationally modified residue), and DNA_BIND (binding site residues to DNA) at protein-protein interfaces in COSMIC in comparison with 1000G. Notice that features with a low percentage of nsSNV produce a less reliable result.
